# Case report of ischemic stroke in a child secondary to neuroborreliosis

**DOI:** 10.1007/s10072-025-08296-3

**Published:** 2025-06-19

**Authors:** Dawid Zakrzewski, Mateusz Tomkiewicz, Wiktoria Kubziakowska, Marta Zawadzka, Jakub Szymarek, Maria Mazurkiewicz-Bełdzińska

**Affiliations:** Department of Developmental Neurology, Medical University of Gdansk, 80-210, Gdansk, Poland

**Keywords:** Stroke in children, Ischemic stroke in children, Neuroborreliosis, Childs neuroborreliosis, Lyme disease

## Abstract

**Background:**

Neuroborreliosis is a neurological manifestation resulting from bacteria of the *Borrelia burgdorferi* sensu lato complex infection. This condition presents with a range of heterogeneous symptoms affecting the central and peripheral nervous systems, manifesting with varying frequency in adults and children. In rare cases, the infection may lead to ischemic stroke, however the pathogenesis remains incompletely understood due to its infrequent occurrence. The development of ischemic stroke in these patients is likely mediated by arterial inflammation, leading to critical narrowing of blood vessels and subsequently to ischemia.

**Case presentation:**

This paper presents the case of a 16-year-old patient diagnosed with ischemic stroke. The patient was treated with enoxaparin until exclusion of cardioembolism and vascular dissection. After excluding other potential causes, neuroborreliosis was considered highly probable due to a history of multiple tick bites and elevated titers of antibodies against *Borrelia burgdorferi* in both blood serum and cerebrospinal fluid. Therefore, the patient received antibiotic therapy. In long-term follow-up, he has shown significant improvement, returning to his pre-stroke level of function.

**Conclusions:**

The lack of literature on the subject contributes to the diagnostic and therapeutic challenges in affected patients. The patient's condition improved as a result of early recognition and prompt initiation of treatment and rehabilitation. It is becoming increasingly important to differentiate paediatric patients with a diagnosis of ischemic stroke also with regard to neuroborreliosis in endemic regions.

## Background

Lyme disease is the most common vector borne disease in Europe with its prevalence largely dependent on geographic location [1]. It is caused by a complex of bacteria from the *Borrelia burgdorferi* sensu lato group, which includes six species pathogenic to humans: *B. burgdorferi*, *B. garinii*, *B. afzelii*, *B. spielmanii*, *B. bavariensis*, and *B. mayonii*, the first five of them are found in Europe [2]. Transmission to humans occurs by a vector—the Ixodes tick, which introduces the bacteria into the human bloodstream through a bite [3].

Symptoms of Lyme disease may manifest early or late in the course of infection and can affect the skin, heart, joints and nervous system, with nervous system involvement known as neuroborreliosis [4]. Early neuroborreliosis, which typically develops within six months of infection, is more common and may present in diverse forms, including painful meningitis, unilateral or bilateral facial paresis, cranial nerve inflammation, nerve plexus inflammation, or single nerve inflammation. In contrast, late neuroborreliosis develops after six months and it is less common and may present as peripheral neuropathy, cerebral vasculitis, chronic progressive Lyme encephalitis or encephalomyelitis. Symptoms of late neuroborreliosis include tetraspastic syndrome, spastic-ataxic gait disorders and urinary dysfunction [5, 6].

The frequency and presentation of clinical symptoms vary with age. In adults, meningitis is most frequently observed, whereas in children cranial nerve involvement is more prevalent, most commonly affecting the facial nerve [7]. Additionally, in young children atypical symptoms such as loss of appetite, fatigue and mood changes may occur [8].

Differences have been observed in the anatomical distribution of tick bites between children and adults. In pediatric patients, tick bites are more commonly located on the ears, neck and head regions closer to the central nervous system (CNS), which is associated with a higher risk of developing neuroborreliosis [9].

A rare, but serious complication of neuroborreliosis in children is ischemic stroke, which may result from inflammatory processes in cerebral vessels leading to critical narrowing of blood flow [10]. This paper presents the case of a patient, who experienced an ischemic stroke in the course of neuroborreliosis.

## Case Presentation

A 16-year-old boy, with no significant prenatal or perinatal history, diagnosed with autism spectrum disorder, was urgently admitted to the Developmental Neurology Clinic at the University Clinical Center in Gdańsk due to a sudden loss of muscle strength in his right limbs, raising suspicion of stroke.

At 2 years and 8 months of age, the boy was diagnosed with autism, along with epilepsy characterized by myoclonic-astatic seizures, which was successfully managed with levetiracetam and clonazepam pharmacotherapy, resulting in complete seizure control. He is currently not taking any antiepileptic medications.

Upon admission, the patient was in moderate general condition with stable circulatory and respiratory functions and was able to communicate in verbal and logical way. According to his parents, during a school trip on the morning of September 6, 2023, he reported gait disturbances. On the day of admission (September 7, 2023), at approximately 6:00 a.m., they noted further weakening of muscle strength in his right limbs, which rendered him unable to move independently. Neurological examination revealed slurred speech (a pre-existing condition), right-sided hemiparesis (right upper limb—2/5 and right lower limb—3/5) with a positive Babinski sign on the right side.

**Fig. 1:**
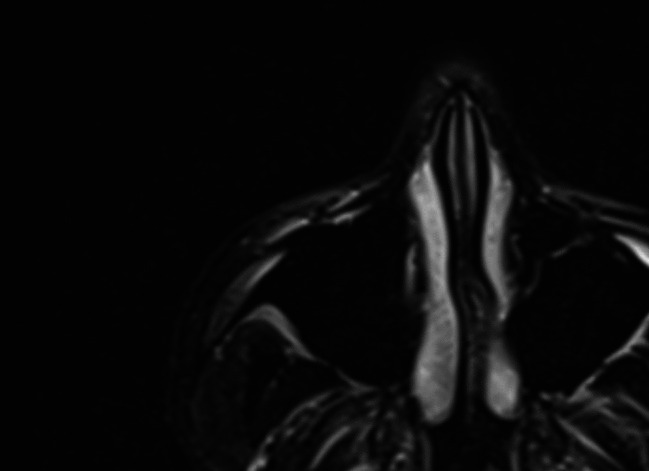
MRI of the head image. In the ventral central-left-side part of the medulla oblongata, in its middle section, an area of ​​increased signal with features of diffusion restriction (size 9 × 6 mm) corresponding to an ischemic focus is visible

During hospitalization, magnetic resonance image (MRI) of the head with vascular imaging was conducted, revealing an area of hyperintensity with diffusion restriction in the ventral, central-left-lateral portion of the medulla oblongata, in its midsection. The lesion, pyramidal in shape and measuring up to 9 × 6 mm, was consistent with acute ischemia (Fig. 1). The patient was diagnosed with ischemic stroke and treatment with low-molecular-weight heparin (2 × 40 mg) was initiated, with the intention of shifting to antiplatelet therapy after exclusion of cardioembolism and vascular dissection. Alongside, the recommendation included his rehabilitation.

As part of the diagnostic workup, systemic lupus erythematosus, other rheumatological diseases, thrombophilia and secondary coagulation abnormalities were ruled out. No vascular abnormalities were observed in angioMRI imaging. The patient was also referred for cardiology consultation and underwent transthoracic and transesophageal echocardiography, which revealed no abnormalities. Additionally, Doppler ultrasound of the carotid arteries and contrast-enhanced transcranial Doppler ultrasound were performed, both showing normal flow parameters.

(collections of the Department of Radiology, University Clinical Center in Gdańsk).

**Fig. 2:**
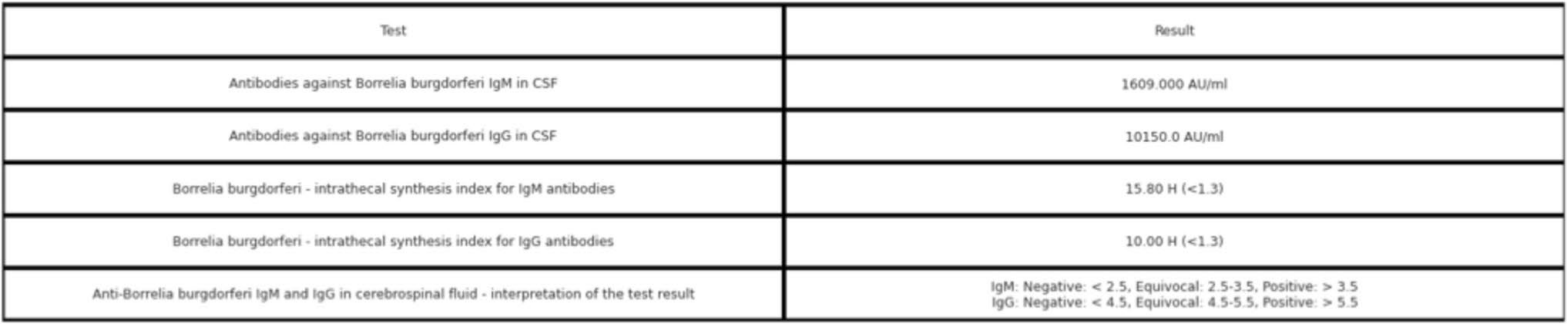
Serodiagnosis of Lyme disease with CFS (collections of Central Clinical Laboratory, University Clinical Center in Gdańsk)

During hospitalization, elevated antibody levels against *Borrelia burgdorferi* were detected in the patient’s blood serum: Immunoglobulin M (IgM) 344.3 AU/ml (N < 18 AU/ml) and Immunoglobulin G (IgG) 6550 AU/ml (N < 10 AU/ml). The Western Blot test returned positive, despite that the patient did not present other typical symptoms of *Borrelia burgdorferi* infection, such as joint or skin changes or cardiac symptoms. Upon further questioning, it was revealed that the patient had experienced multiple tick bites while exposed to grassy and forested areas over the summer. Due to remaining diagnostic uncertainties, a lumbar puncture was performed to assess antibody levels in the cerebrospinal fluid (CSF). Antibody titers in the cerebrospinal fluid were measured at 10,150 AU/ml for IgG and 1,609 AU/ml for IgM (Fig. 2). Additional cerebrospinal fluid analysis showed pleocytosis (142 cells/µl), elevated protein levels (1.43 g/l) and a lymphocyte count of 81%.

The patient was started on ceftriaxone at a dose of 2 g daily for 28 days and was transferred to the Infectious Disease Hospital in Gdynia for ongoing treatment. In light of the recent stroke, high antibody titers against *Borrelia burgdorferi*, and the exclusion of other potential causes of CNS ischemia, a probable diagnosis of stroke secondary to neuroborreliosis was established. Following the diagnosis, it was recommended to withdraw the administration of anticoagulant therapy on its 20th day.

According to both the patient and his parents, following discharge from the Developmental Neurology Clinic, muscle strength in his right upper and lower limbs improved. He regained independent ambulation and continued with rehabilitation.

**Fig. 3:**
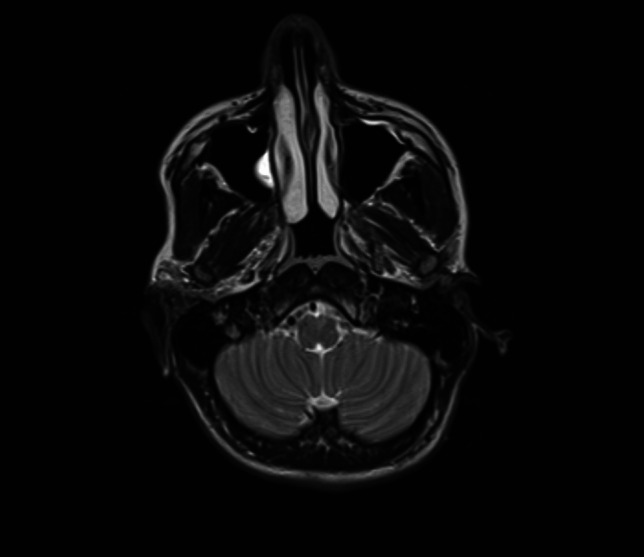
MRI of the head image. Small area (size 5 × 2.5 mm) of ​​discreetly increased T2WI/FLAIR signal, without diffusion restriction, without pathological contrast enhancement corresponding to gliosis after an ischemic episode

At a follow-up visit on August 22, 2024, the patient exhibited slightly reduced muscle strength in the right upper and lower limbs (4/5), along with increased muscle tone and a positive Babinski sign on the right side. His daily functioning has not significantly changed from his pre-stroke level, though he reports minor challenges with fine motor tasks. Follow-up MRI showed a small area of gliosis corresponding to the ischemic lesion site Fig. 3.

(collections of the Department of Radiology, University Clinical Center in Gdańsk).

**Fig. 4:**
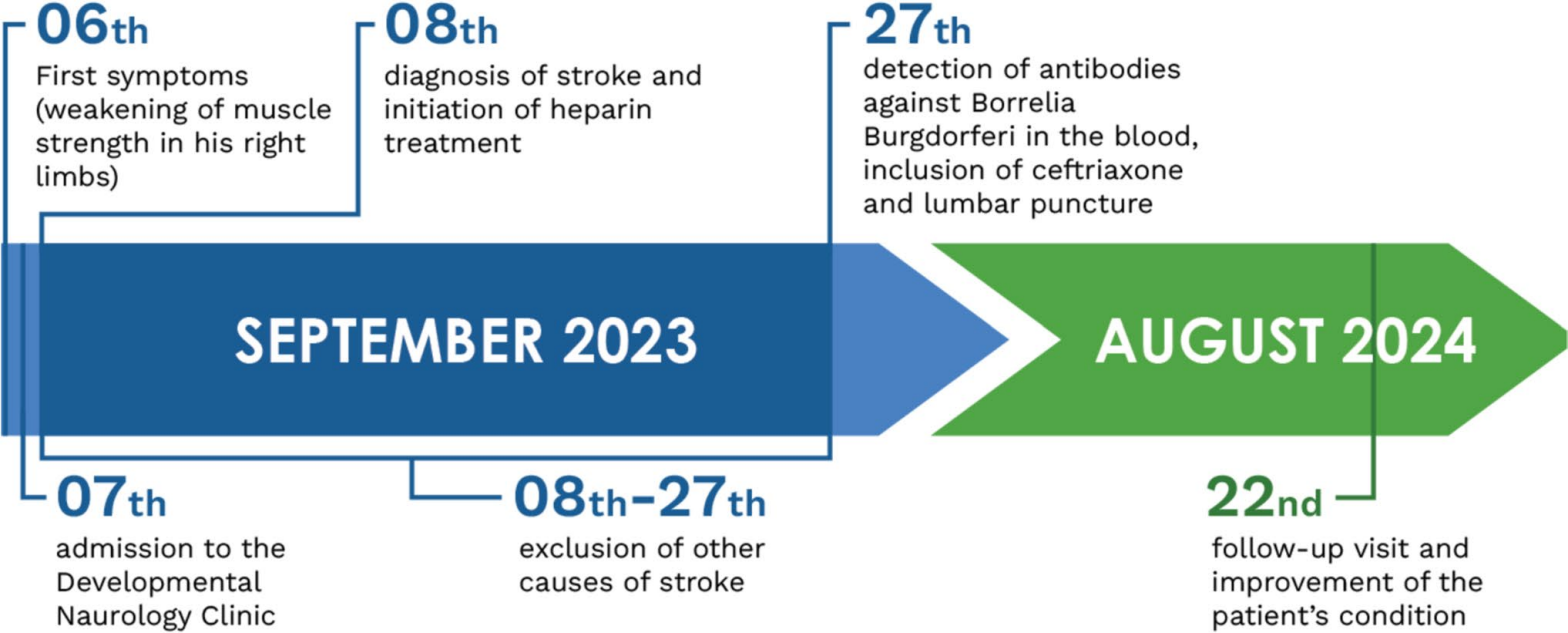
Summary timeline

The patient and his parents consented to share their experience with the authors, providing insight into the course of the illness and treatment outcomes, and agreed to the publication of the diagnostic and therapeutic process described above. They expressed satisfaction with the medical care provided and are pleased with his recovery and return to pre-stroke functioning Fig. 4.

## Discussion

Stroke is considered a very rare manifestation of neuroborreliosis [11]. A PubMed database search using the query"stroke AND (neuroborreliosis OR borreliosis OR Lyme disease OR Borrelia) AND case report"yields only 59 results from 1994 to 2024, and large-scale studies assessing the frequency of neuroborreliosis-related cerebrovascular events remain lacking. Mironova et al. identified the prevalence of ischemic stroke/TIA among adult borreliosis-positive patients at Helsinki University Hospital to be 0.6% [12], while an analysis of a population of 229 Swiss children with ischemic stroke or vasculitis identified four cases (1.7%) potentially linked to Lyme Neuroborreliosis (LNB) [13]. Among the different genospecies, *Borrelia burgdorferi* sensu stricto is the primary cause of Lyme disease in the USA, whereas *B. garinii* and *B. afzelii* are more common etiological agents in Europe, with the latter being considered more neurotropic. This may account for the more severe course of LNB in Europe. Additionally, *Ixodes persulcatus*, a tick species responsible for transmitting *B. garinii* but not *B. burgdorferi* sensu stricto, is found in northern Asia [14, 15]. As LNB-related stroke and LNB, in general, appear more prevalent in Europe than in the USA, with generally more severe clinical course, information on the patient's country of residence may be valuable [14, 16, 17]. Since our patient was residing in Poland, the natural habitat of B. burgdorferi vector—the Ixodes ricinus tick species, LNB as underlying cause of the stroke was also considered [18]. History or presence of additional infection symptoms might support the diagnosis of LNB related arterial ischemic stroke (AIS), but their low sensitivity hinders their clinical relevance. Incidence of erythema migrans, a symptom often considered pathognomonic to Lyme disease is estimated to be around 80% in affected patients [19]. In a review of published cases, just 26% of patients with LNB-related cerebrovascular events had a history of erythema migrans [16], which might explain the lack of this clinical manifestation in our patient despite extensive tick-bite history. The discrepancy might stem from the uncertain and often long time distance between the prodromal phase of the infection and its cerebrovascular manifestations.

The exact mechanism of LNB-related stroke remains unknown; however, evidence suggests the role of infectious cerebral vasculitis in its pathogenesis. Inflammation of cerebral vessels has been proven to be a possible consequence of several bacterial, viral, fungal and parasitic infections. The vascular damage leading to inflammatory response may occur as a result of direct infection of endothelium, release of toxic mediators, presence of septic emboli, mycotic aneurysms or inflammatory exudate. Vascular insult leads to further inflammation as well as morphological changes to vessel walls [20, 21]. CNS invasion by *Borrelia burgdorferi*, similar to another spirochete, *Treponema pallidum* (the causative agent of syphilis), may lead to inflammation of cerebral vessels, which can manifest as endarteritis obliterans in small vessels or inflammation in medium- or large-sized vessels [16, 17, 22, 23]. Pathogen infiltration may occur through cranial nerve pathways, and inflammation in the form of lymphocytic and plasmacytic perivascular infiltrate arising from Borrelia-related meningitis has been highlighted [24, 25]. In a study of Rhesus macaques with induced CNS Borrelia burgdorferi infection, CSF samples showed elevated levels of proinflammatory cytokines, specifically interleukin 8 (IL-8), C–C Motif Chemokine Ligand 2 (CCL2), and C-X-C motif Chemokine ligand 13 (CXCL13), which are potent immune cell attractants [25].

In addition to Borrelia-induced vasculitis, the direct prothrombotic effect of the infection itself might also play an important role in the cerebrovascular manifestations of LNB. Infectious state is linked to cytokine and complement-mediated endothelial injury, elevated markers of thrombosis, and finally—thrombotic clinical manifestations, such as stroke, as seen in SARS-COV-2 infection [26, 27], as well as in other infectious diseases [28, 29]. This hypothesis is further supported by laboratory evidence suggestive of recent infection in our patient (high CSF and serum IgM-levels).

Epidemiological data emphasize the role of vasculitis and infections as primary risk factors for pediatric AIS [30, 31]. In a longitudinal study, Winter et al. reported that in patients with neuroborreliosis-related vasculitis, posterior circulation was affected more frequently than in the non-borreliosis stroke population (60.8% and 27.5%, respectively) [24]. In a systematic review of published cases, Garkowski et al. found that 75.6% of cerebrovascular manifestations of Lyme disease involved the posterior circulation, with the basilar artery being the second most commonly affected vessel [16]. Consistent with these findings in the described above case report, the patient experienced a stroke specifically involving the posterior circulation as well. When diagnosing LNB-vasculitis, imaging studies, such as computer tomography-angiography (CTA), magnetic resonance-angiography (MRA) or digital subtraction angiography (DSA) are standard diagnostic methods employed to assess morphological changes in cerebral vasculature, such as stenosis or lumen dilation. However, as size of affected vessels decreases, the likelihood of false negative study outcome increases, resulting in relatively low sensitivity of CTA, MRA or DSA in detecting small vessel vasculitis [32, 33]. Since LNB-vasculitis can affect small vessels, radiological findings alone cannot rule out the condition [16, 34, 35], which might explain lack of evidence of vasculitis in this particular clinical case, as well as in other case reports [36]. Additionally no Digital Subtraction Angiography was performed.

To make a solid diagnosis of neuroborreliosis according to the European Federation of Neurological Societies (EFNS) Guidelines for the Diagnosis and Management of European Lyme Neuroborreliosis, the following diagnostic criteria must be met: neurological symptoms suggestive of neuroborreliosis unexplained by other obvious causes, cerebrospinal fluid pleocytosis, intra-thecal production of antibodies against B. burgdorferi. In this particular clinical case, the patient's symptoms could not be explained by other causes of ischaemic stroke, which are the most common in the population, and the diagnosis of neuroborreliosis was made by fulfilling both laboratory conditions [37–39].

Our patient was not eligible for reperfusion therapy (the time since symptom onset was greater than 4.5 h, and there was no evidence of large-vessel occlusion) [40–42]. The patient was treated with enoxaparin until exclusion of cardioembolism and vascular dissection with the intention of following shift to antiplatelet therapy. It must be emphasised that the main goal of antiplatelet or anticoagulant therapy is to prevent recurrence of stroke[43]. The long-term antiplatelet therapy was subsequently abandoned in this patient upon identification of the cause and targeted therapy (antibiotic therapy). Nevertheless, intravenous thrombolysis (IVT) and endovascular thrombectomy (EVT), commonly used in adult acute AIS, are also considered in pediatric stroke cases.

Reports of reperfusion therapy in pediatric LNB-related stroke are scarce. One case of a 13-year-old patient with LNB-related AIS treated with IVT showed a favorable outcome, with a Pediatric National Institutes of Health Stroke Scale (PedNIHSS) score reduction from 10 at 24 h post-treatment to 2 at a nine-month follow-up [44].

Retrospective studies and meta-analyses also highlight high-recovery potential in EVT-treated pediatric-AIS patients [45, 46]. A 6-year-old patient with LNB-related AIS with severe symptoms—including coma, anisocoria, hemiparesis, and decerebrate posturing—was successfully treated with EVT, achieving a thrombolysis in cerebral infarction (TICI) scale 3 recanalization and a PedNIHSS score reduction from 32 at admission to 0 on day 10 [44].

Case-based data, retrospective studies, and expert opinions support cautious optimism regarding reperfusion therapies in childhood AIS, regardless of whether the etiology is infectious. However, the need for large-scale randomized clinical trials remains evident.

Our patient also received ceftriaxone which resulted in a good clinical response with remission of most of the neurological symptoms. This is consistent with other case reports of LNB-AIS ceftriaxone- or doxycycline-treated patients [16, 36, 47].

## Conclusion

Although neuroborreliosis is an uncommon cause of stroke, it is a potential differential diagnosis for cerebrovascular events, particularly in paediatric populations and among patients presenting with clinical features suggestive of posterior circulation infarction, especially those residing in Lyme disease–endemic regions. This case further highlights the diagnostic challenges posed by the potential coexistence of distinct neurological conditions.

## Data Availability

The clinical data comes from the collection of the Department of Developmental Neurology, University Clinical Center in Gdańsk, and has been anonymized in the above work.

## Abbreviations

CNS: Central nervous system
MRI: Magnetic resonance image
Ig: Immunoglobulin
CSF: Cerebrospinal fluid
LNB: Lyme Neuroborreliosis
AIS: Arterial ischemic stroke
IL-8: Interleukin 8
CCL2: C-C Motif Chemokine Ligand 2
CXCL13: C-X-C motif Chemokine ligand 13
CTA: Computer tomography-angiography
MRA: Magnetic resonance-angiography
DSA: Digital subtraction angiography
EFNS: European Federation of Neurological Societies
IVT: Intravenous thrombolysis
EVT: Endovascular thrombectomy
PedNIHSS: Pediatric National Institutes of Health Stroke Scale
TICI: Thrombolysis in cerebral infarction scale
